# Leveraging compact convolutional transformers for enhanced COVID-19 detection in chest X-rays: a grad-CAM visualization approach

**DOI:** 10.3389/fdata.2024.1489020

**Published:** 2024-12-16

**Authors:** Aravinda C. V, Sudeepa K. B, S. Pradeep, P. Suraksha, Meng Lin

**Affiliations:** ^1^Department of Computer Science and Engineering, NITTE Mahalinga Adyantaya Memorial Institute of Technology, NITTE Deemed to Be University, Karkala, Karnataka, India; ^2^Department of Computer Science and Engineering, Government Engineering College, Chamarajanagar, Karnataka, India; ^3^Department of Computer Science and Engineering, Vidhya Vardhaka College of Engineering, Mysore, Karnataka, India; ^4^Department of Electronic and Computer Engineering (The Graduate School of Science and Engineering), Ritsumeikan University, Kusatsu, Shiga, Japan

**Keywords:** deep learning, COVID-19 detection, chest X-ray analysis, convolutional neural networks, grad-CAM visualization, data augmentation, machine learning algorithms, public health informatics

## 1 Introduction

The evolution of Deep Learning (DL) methodologies has significantly enhanced the field of medical Imaging, particularly in the interpretation of chest radiographs (CXRs). Among these advancements, Convolutional Neural Networks (CNNs) have emerged as a paramount technology for processing and Classifying CXR images and demonstrating exceptional proficiency in detecting COVID-19-related signs (Perumal et al., 2020). Although Reverse Transcription Polymerase Chain Reaction (RT-PCR) tests surpass CXRs in accuracy and reliability for virus detection, the latter remains an ubiquitous tool in clinical practice (Giri and Rana, 2020). RT-PCR excels in early detection capabilities, enabling prompt treatment initiation, and uniquely identifies the virus in asymptomatic individuals through the analysis of: Saliva samples, throat samples, and nasal passage samples, demonstrating superior performance over CXR evaluations in these aspects.

A recent investigation employed a Convolutional Neural Network (CNN) model to distinguish between:

- Normal chest radiographs.
- Pneumonia-infected.
- COVID-19 affected individuals.

The CNN model, trained on an extensive dataset comprising images from:

- Patients diagnosed with pneumonia.
- Those testing positive for COVID-19.

Healthy subjects achieved a remarkable precision rate of 97.6% in identifying COVID-19 cases on chest radiographs (CXR). This exceptional level of precision surpasses that of traditional CXR diagnostic techniques, demonstrating the potential of deep learning algorithms to improve diagnostic accuracy (Wang and Lin, 2020). In the current healthcare landscape, the surge in demand for intensive care units (ICUs) has exposed the capacity constraints of healthcare systems in several developed countries. The influx of patients suffering from COVID-19-induced pneumonia into the ICU underscores this pressing challenge, highlighting the need for effective diagnostic tools and strategies to manage the burden on healthcare resources (Kermany et al., 2018).

The system employs relational feature intelligence to analyze and interpret the interactions among various elements within an image, enabling the assessment of:

- Spatial relationships.
- Dynamics between different components.

This capability facilitates the evaluation of:

- Tumor-tissue interactions.
- Inter-organ relationships (e.g., heart-lung interactions) which is crucial for diagnosing conditions like:
  ∘ Heart failure.
  ∘ Pulmonary embolism.

The relational feature intelligence of the system enables the analysis of complex interactions within medical images, providing valuable information on spatial relationships and dynamics between different elements (Ai et al., 2020; Ng et al., 2020; Kong and Agarwal, 2020). This capability is particularly beneficial in:

- Oncology (accurate diagnosis and treatment planning).
- Cardiovascular and pulmonary diseases (diagnosing conditions like heart failure and pulmonary embolism).

**Figure 1:**
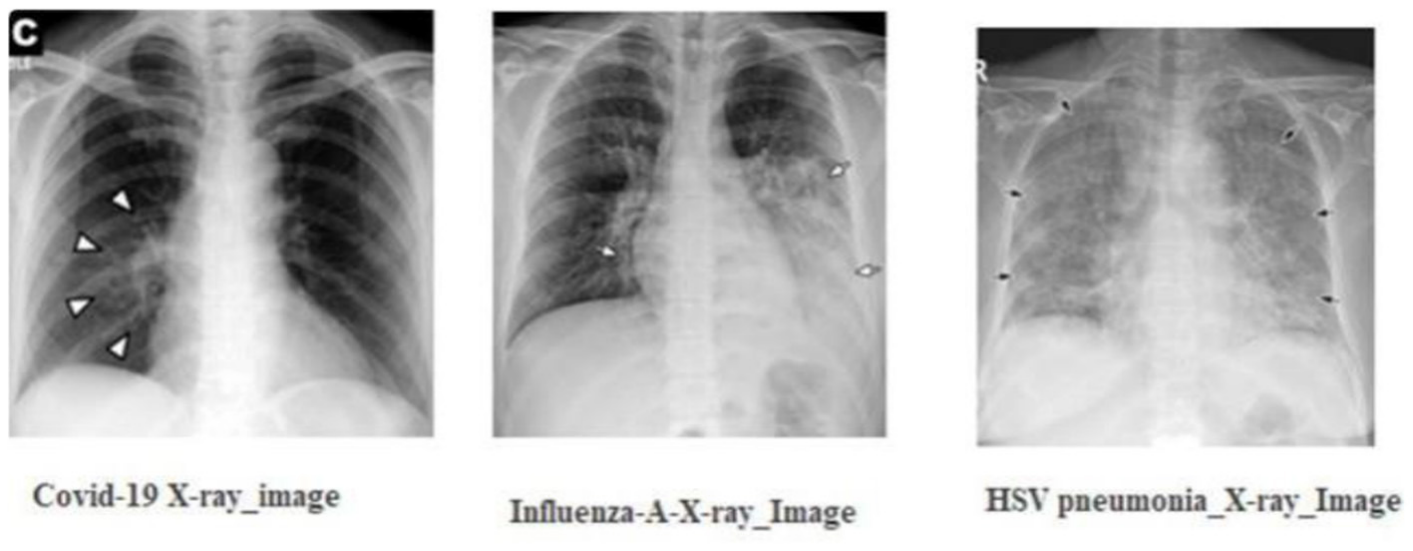
The radiographic characteristics indicative of COVID-19, showing bilateral and lower-zone dominant ground-glass opacities (GGOs) along with consolidations.

As depicted in Figure 1, the radiographic features characteristics of COVID-19 typically encompass:

- Bilateral and lower-zone dominant ground-glass opacities (GGOs).
- Consolidations, predominantly peripheral in distribution.
- Interlobular septal thickening.
- Pleural effusions.

In contrast, viral pneumonia caused by non-SARS-CoV-2 viruses, such as influenza-A, tends to exhibit:

- Unilateral, central, and upper-zone dominant GGOs and consolidations Using these distinctions, medical experts advocate the concurrent use of:
  ∘ Chest radiography.
  ∘ Nucleic acid amplification tests

as a primary diagnostic strategy for early identification of this novel pneumonia strain.

The primary objective of this examination is to detect the presence of the virus within a patient, which is achieved through the analysis of specific biomarkers (Choe et al., 2019). To augment this assessment, medical practitioners often employ a multimodal diagnostic approach, incorporating additional methodologies such as:

- Antibody tests,
- Antigen tests, and
- Radiographic analysis via chest X-rays

to verify infection and facilitate accurate diagnosis. However, it is crucial to acknowledge that these supplementary tests may not always yield precise outcomes, highlighting the necessity for their combined application with other diagnostic techniques to ensure a definitive diagnosis.

A significant limitation of this specific examination is the considerable:

- Processing time
- Expenses

associated with its execution, which may hinder its applicability in resource-constrained settings.

To minimize diagnostic errors, healthcare professionals are advised to utilize automated imaging analysis software, powered by artificial intelligence (AI), when interpreting X-ray photographs. These advanced tools excel at identifying complex patterns within images, thereby improving diagnostic accuracy. Additionally, to maintain a high level of diagnostic precision, medical staff should take periodic breaks to avoid fatigue and seek second opinions from colleagues to ensure reliability and accuracy. Innovations in diagnostic methodologies, such as the integration of AI and machine learning (ML) technologies, can significantly reduce both the time and financial costs associated with diagnostic processes, enhancing efficiency and reliability. Specifically, AI and ML can accelerate and refine disease diagnosis by analyzing medical data. In addition, digital biomarkers, such as data from wearable technologies, offer real-time information, enabling the early detection and monitoring of various health conditions. The synergy of ML and AI has facilitated the development of sophisticated algorithms capable of detecting and diagnosing diseases with remarkable precision. These advancements have transformed the diagnostic landscape, enabling healthcare professionals to make more accurate and timely diagnoses (Simonyan and Zisserman, 2015). Artificial intelligence (AI)-empowered systems can efficiently process vast datasets, enhancing the capacity for early disease detection and the formulation of customized treatment plans. The advent of telemedicine and remote monitoring tools enables healthcare practitioners to oversee patient health remotely, providing timely interventions when necessary. This approach has been particularly advantageous during the pandemic. Pre-trained models can address complex issues with speed and accuracy, offering a significant advantage in medical diagnostics. Leveraging pre-trained models reduces the time and resources required for training a model from scratch, while enhancing precision. These models facilitate knowledge transfer across different domains, enabling more efficient and accurate solutions. The Grad-CAM algorithm serves as a visualization tool, elucidating the decision-making process of convolutional neural networks (CNNs). By utilizing gradients pertaining to a selected target class, Grad-CAM generates a localization map highlighting pivotal areas within an image critical for identifying a specific concept. This study introduces a comprehensive transfer model for synchronous COVID-19 detection and visualization of affected areas using X-ray imaging (He et al., 2016).

Unlike prior research, our work focuses on COVID-19 detection via X-ray images. Given the pressure on healthcare systems, it is crucial to leverage every available resource efficiently. Integrating the Grad-Cam localization feature aids in identifying COVID-19 and assessing severity, assisting in determining whether immediate healthcare intervention is needed (Selvaraju et al., 2016). In our investigation, we leveraged a diverse range of pre-trained models for the classification task, including:

- ResNet34.
- ResNet50.
- EfficientNet-B4.
- EfficientNet-B5 architectures.

The incorporation of Grad-CAM into our methodology enabled the precise identification of affected regions, with the EfficientNet architecture being utilized for its exceptional:

- Efficiency.
- Effectiveness.

The utilization of pre-trained models offers significant advantages, primarily due to their pre-learned weights, which make them exceptionally valuable even when working with limited datasets. This constitutes the primary benefit of integrating pre-trained models into our investigative approach. Additionally, the employment of pre-trained models reduces the:

- Computational power
- Resources required for training

making them a computationally efficient option.

To address the variability in light intensity of the captured images, we applied the Contrast Limited Adaptive Histogram Equalization (CLAHE) technique to enhance image quality, ensuring a more accurate analysis. This preprocessing step enabled the:

- Improvement of image contrast
- Reduction of noise

ultimately leading to more reliable results.

## 2 Contribution of the work

In this study, we leveraged publicly accessible datasets of X-ray images as the primary experimental medium. The architecture of our experiment is designed as an end-to-end system, eliminating the need for manual feature extraction or selection, thereby streamlining the process for enhanced efficiency and effectiveness. We employed the Grad-CAM technique in conjunction with a Convolutional Neural Network (CNN) model to improve the diagnostic accuracy of our system. The integration of Grad-CAM enables the visualization of feature importance, allowing us to identify the most relevant regions in the X-ray images for diagnostic decision-making. By utilizing this approach, we aimed to develop a robust and accurate diagnostic system, capable of automatically detecting, and localizing abnormalities in X-ray images, thereby assisting clinicians in making informed decisions.

### 2.1 Methodological framework

The core of our methodological approach integrates the Gradient-weighted Class Activation Mapping (Grad-CAM) technique with Convolutional Neural Network (CNN) architectures, aiming to refine diagnostic precision through the following formulation:


(1) $$\begin{array}{l} L_{Grad-CAM}=ReLU\left(\sum_{k}a_{k}^{e}A^{k}\right) \end{array}$$

where *L*_Grad − CAM_ represents the localization map highlighting regions of interest, $a_{k}^{e}$ denotes the weights for the *k*-th feature map *A*^*k*^ contributing to a target class *c*, and ReLU ensures the activation map focuses on features positively influencing the class prediction.

For classification purposes, we employed a selection of pre-trained models:

- ResNet34 and ResNet50 and
- EfficientNet-B4 and EfficientNet-B5,

fine-tuned to adapt their learned representations to our specific task. This process exploits the models' preexisting knowledge, significantly economizing on computational resources and training time.

The Grad-CAM heatmap is computed using the equation: L Grad-CAM = ReLU (∑ k ${\text{a}}_{\text{k}}^{\text{e}}$ A^k^). This equation is crucial in visualizing the regions of the input image that significantly influence the model's decision-making process. In our study, we utilize the feature maps A k from the last convolutional layer of the CNN and importance weights a k e derived from the gradients of the predicted class score with respect to A^k^. These weights represent the contribution of each feature map to the prediction. The ReLU function ensures that only positive contributions are considered, highlighting the most relevant regions for class prediction. This equation enables the generation of Grad-CAM heatmaps, which are overlaid on original chest X-rays to help clinicians identify critical regions that influence the model's classification.

### 2.2 Analytical approach

Our investigation scrutinizes the influence of the characteristics of the data set and image processing techniques on the precision of disease detection. This entails a dual analysis approach:

#### 2.2.1 Dataset analysis


(2) $$\begin{array}{l} \Delta_{accuracy}=f\left(Dataset_{balance},\;{\;Image}_{enhancement}\right) \end{array}$$

where Δ accuracy measures the change in diagnostic accuracy as a function of dataset balance and image enhancement techniques.

#### 2.2.2 Image enhancement

The employment of image enhancement, particularly for X-ray and CT-Scan images, was operationalized through the application of Contrast Limited Adaptive Histogram Equalization (CLAHE), aiming to ameliorate image quality for more accurate diagnostic interpretation.

#### 2.2.3 Justification of using these models

When selecting models for this research, several factors were considered to ensure the best choices for the task at hand. First, computational feasibility was evaluated. While models like DenseNet and Inception are powerful, they require significant computational resources without offering substantial accuracy improvements over other options. So, more efficient architectures that strike a balance between performance and computational demands were selected. The selected models are known for their ability to adapt well to different datasets, which is crucial given the variations in chest X-ray imaging conditions. By considering these factors, the research offers the best combination of accuracy, efficiency, and generalizability.

### 2.3 Hyperparameter tuning and sensitivity analysis

#### 2.3.1 Sensitivity analysis

To evaluate the model's robustness, sensitivity analyses were performed on key hyperparameters.

#### 2.3.2 Learning rate sensitivity

A significant performance drop was observed when the learning rate deviated from the optimal value of 10^−3^, highlighting its crucial role in convergence. To address this, a learning rate scheduler was employed to dynamically adjust the learning rate upon validation loss plateauing.

#### 2.3.3 Batch size sensitivity

Increasing the batch size to 64 negatively impacted performance due to poorer gradient estimates on limited GPU memory. Conversely, smaller batch sizes increased training time.

#### 2.3.4 Dropout rate sensitivity

Dropout rates below 0.2 led to overfitting, characterized by high training accuracy but low validation accuracy. In contrast, dropout rates above 0.5 hindered the learning process.

#### 2.3.5 Optimizer sensitivity

The Adam optimizer demonstrated robustness for the dataset used in this study, showing less sensitivity to small learning rate changes compared to SGD.

## 3 Literature survey

The collaborative efforts of Eduardo A. Soares, Plamen P. Angelov, and Sarah Biaso have culminated in a significant contribution to the field of medical imaging for infectious diseases. They have developed and made publicly available a comprehensive dataset of CT scans specific to SARS-CoV-2, enabling the advancement of diagnostic capabilities.

The team crafted an innovative algorithm, meticulously training it on CT scans from both:

- Confirmed SARS-CoV-2 positive patients.
- Patients without the infection.

This training phase was followed by a rigorous testing phase, where the algorithm's efficacy was validated on a distinct dataset encompassing CT scans from individuals with and without SARS-CoV-2 infection.

The outcomes of this testing phase demonstrated the algorithm's precision in accurately detecting SARS-CoV-2 infection, highlighting its potential utility in enhancing diagnostic processes within clinical settings. This contribution has the potential to significantly impact the field of medical imaging for infectious diseases, improving patient outcomes and streamlining diagnostic procedures (Soares et al., 2020). In a groundbreaking study, Sara Haseli and Nastaran Khalili have significantly advanced our understanding of COVID-19 pneumonia through comprehensive radiological analysis of chest CT imaging. Their research elucidated distinct hallmark features characteristic of the condition, including:

- Bilateral ground-glass opacities.
- Consolidation.
- Interlobular septal thickening.

These findings align with established diagnostic criteria for COVID-19 pneumonia, providing critical insights into the disease's pulmonary manifestations.

Furthermore, their investigation revealed additional complications in a subset of patients, including:

- Pleural effusions.
- Lymphadenopathy.
- Pulmonary embolism.

These findings broaden our understanding of the disease's impact on pulmonary structures, underscoring the importance of vigilant radiological monitoring and timely diagnosis (Haseli et al., 2020). In terms of pulmonary involvement, the posterior segment of the left lower lobe (LLL) was identified as the most frequently affected segment, exhibiting a high propensity for involvement. Additionally, significant instances of involvement were observed in the right middle lobe (RML) and the right lower lobe (RLL), suggesting a bilateral distribution of pulmonary affliction. Upon analyzing the data based on lobar distribution, the LLL exhibited the highest frequency of affliction, with the RLL and RML closely following in prevalence, indicating a relatively even distribution of pulmonary involvement across the lobes (Li et al., 2020a).

Upon examining the demographics of age and gender in relation to chest CT imaging outcomes, a notable pattern emerged, suggesting a gender-specific predilection for lobar involvement. Male patients exhibited a significant propensity for left lower lobe (LLL) involvement, whereas female patients demonstrated a tendency toward more frequent involvement of the right lower lobe (RLL). Further analysis revealed a distinct age-related pattern, with the left lower lobe (LLL) being predominantly affected in the older population (65 years). In contrast, the right lower lobe (RLL) showed a higher incidence of involvement among younger individuals (<65 years). These findings suggest that age and gender may play a role in determining the lobar distribution of pulmonary involvement (Narin et al., 2021).

Wang et al. developed a cutting-edge algorithm for the detection of COVID-19 pneumonia via chest CT image analysis, leveraging an adapted Inception transfer-learning framework. The algorithm's performance was rigorously validated through both internal and external validation processes, demonstrating its efficacy in identifying COVID-19 pneumonia with a high degree of accuracy (Islam et al., 2020).

In their investigative study, Ali Narin and Ceren Kaya put forward three models grounded in convolutional neural network technologyaˆ C”ResNet50, InceptionV3, and InceptionResNetV2aˆ C”for the purpose of identifying coronavirus pneumonia from chest X-ray images. These models underwent rigorous evaluation on a dataset that included both confirmed COVID-19 cases and cases of conventional viral pneumonia, demonstrating the application of advanced deep learning techniques in the differentiation and diagnosis of respiratory illnesses (Saha et al., 2020; Wang et al., 2020; Song et al., 2021). In a pioneering approach, the developed system leveraged a hybrid architecture, synergistically integrating a Long Short-Term Memory (LSTM) classifier with a Convolutional Neural Network (CNN) dedicated to feature extraction and selection. The system's performance was rigorously evaluated using a dataset comprising 421 cases, including 141 instances with features indicative of COVID-19. Following the completion of its training phase, the model demonstrated exceptional performance capabilities, adeptly categorizing images as either COVID-19 positive or negative. The evaluation of the model's efficacy involved the application of 10-fold cross-validation, yielding an impressive accuracy rate of 97.3%. This remarkable achievement underscores the system's potential in medical imaging analysis, showcasing its ability to accurately classify images and support diagnostic decision-making (Mohammad and Abolfazl, 2020; Islam et al., 2020; Alharbi et al., 2022). Extensive validation was conducted on an independent dataset of chest X-ray images, where the proposed model achieved an exceptional accuracy of 97.7%. This remarkable performance demonstrates the model's precision in distinguishing COVID-19 cases, showcasing its potential in medical imaging analysis. The utilized dataset comprised 88 confirmed instances of COVID-19, 101 cases of bacterial pneumonia, and 86 instances classified as normal based on CT scan analyses. Comparative assessments were conducted to evaluate the model's performance relative to traditional frameworks, including Res-Net, Dense-Net, and VGG16. The results underscore the proposed model's enhanced performance, demonstrating its effectiveness through rigorous analysis (Singh et al., 2022; Alharbi et al., 2022a,b).

### 3.1 Multi-modal bone suppression, lung segmentation, and classification approach

This study combines bone suppression and lung segmentation with multi-modal classification to improve COVID-19 detection accuracy. By isolating lung regions, the model reduces noise from surrounding structures, enhancing diagnostic performance. This approach refines lung images, contributing to more accurate identification of COVID-19 in chest X-rays (Li et al., 2020b; Shi et al., 2021).

### 3.2 Comparative study of linear type multiple instance learning techniques

This comparative study investigates various linear multiple instance learning (MIL) models applied to COVID-19 detection in chest X-rays. Analyzing how linear MIL models handle weakly labeled data, the study identifies effective techniques for classifying X-ray images without extensive manual annotations. The findings reveal that certain MIL techniques can efficiently pinpoint COVID-19 indicators, making them suitable for large-scale screenings (Ching et al., 2018; Wang et al., 2017).

### 3.3 Convolutional neural network techniques

CNN-based methods have shown significant promise in detecting COVID-19 from X-ray images. This research explores CNN architectures designed for medical image analysis, fine-tuned to identify COVID-19′s unique patterns in X-rays. Using convolutional layers that capture spatial features, CNNs offer high sensitivity in recognizing infection signs, allowing for robust classification (Wang and Lin, 2020; Apostolopoulos and Mpesiana, 2020).

### 3.4 Machine learning techniques

Beyond deep learning, various machine learning algorithms have been applied to COVID-19 classification in chest X-rays. These techniques include support vector machines (SVMs), decision trees, and ensemble methods. The study highlights the effectiveness of these algorithms in scenarios with limited data, where traditional machine learning methods can outperform deep learning models by leveraging feature extraction and selection methods. These studies demonstrate the potential of AI techniques in enhancing COVID-19 detection accuracy using chest X-rays. Each approach offers unique strengths, and their combination could lead to more effective diagnosis and treatment (Ozturk et al., 2020; Hemdan et al., 2020).

## 4 Materials and methods

### 4.1 Dataset description

While our dataset provides a substantial amount of data for training and evaluating our model, it's important to acknowledge its limitations. The dataset may not fully capture the diversity of COVID-19 cases seen across different populations and imaging conditions, which could impact the generalizability of our model's predictions in real-world clinical settings. For instance, variations in demographics, geographic regions, and imaging equipment could affect the robustness of our model, particularly when applied to data from populations or conditions not represented in the training dataset. To address this limitation, future work should consider incorporating datasets from a broader range of demographics and imaging environments. This would enhance our model's adaptability and effectiveness in diverse healthcare contexts, ensuring that it can provide accurate predictions for a wide range of patients and scenarios. By expanding our dataset in this way, we can increase the confidence in our model's performance and its potential to improve patient outcomes in real-world clinical settings.

The diagnosis of COVID-19 in this study is conducted through the analysis of pulmonary (chest) X-ray images. The dataset is categorized into three primary classes:

- COVID-19.
- Normal.
- Pneumonia.

Sourced from the COVID-19 Radiography Database available on platforms such as Kaggle and Mendeley data, the dataset encompasses a total of 9,300 images, distributed across the classes as follows:

COVID-19: 800 images.
Normal: 2500 images.
Pneumonia: 5,000 images.

## 5 Dataset allocation

The allocation of the dataset for various purposes is segmented as follows:

- Evaluation: 30% of the total dataset.
- Training and Validation: 70% of the total dataset.

Furthermore, within the Training + Validation subset, the images are divided into:

Training: 70%
Validation: 30%

## 6 Visualization

**Figure 2:**
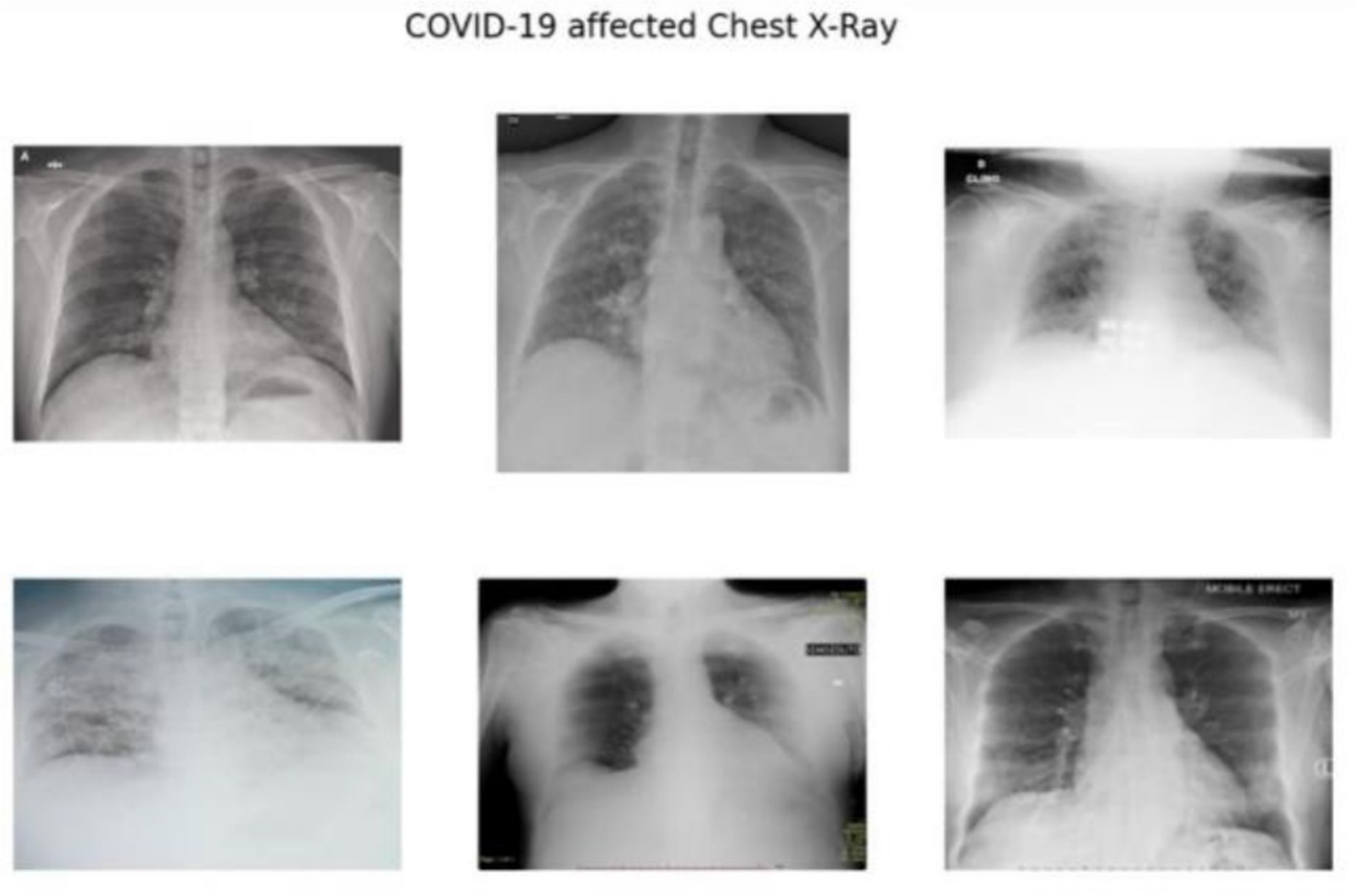
Chest X-ray COVID-19 image samples evaluated using the Kaggle database.

**Figure 3:**
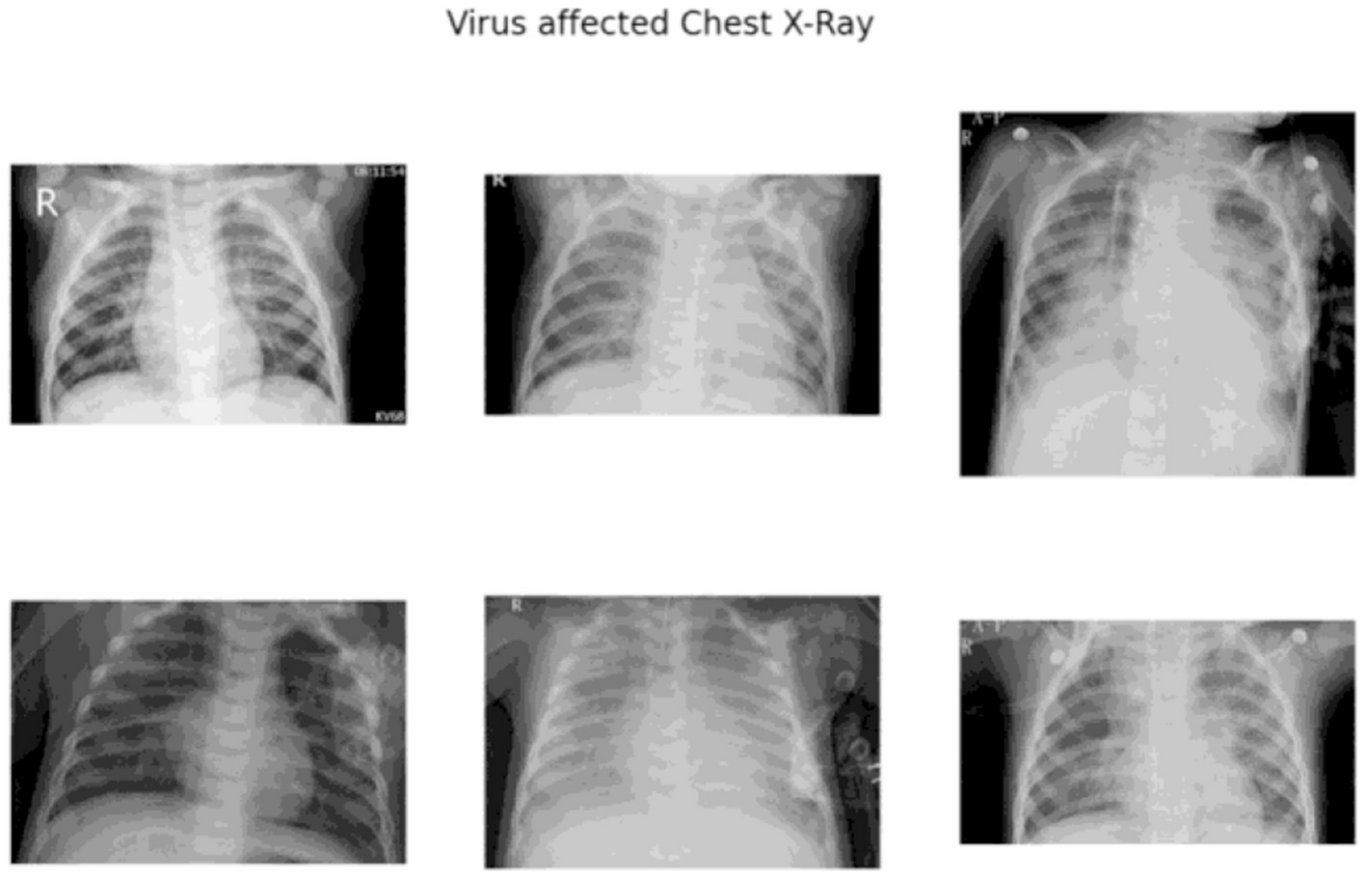
Chest X-ray Pneumonia image samples evaluated using the Kaggle database.

The distribution and representation of all three subsets (COVID-19, Normal, and Pneumonia) are illustrated in Figures 2, 3.

### 6.1 Convolutional layers

Convolutional layers are a fundamental component of Convolutional Neural Networks (CNNs), primarily due to their ability to utilize learnable kernels that perform convolutions across the spatial dimensions of the input data. The convolution operation can be mathematically expressed as:


(3) $$\begin{array}{l} \left(f\;*\;s\right)\left[q\right]=\;\sum_{m=-\infty }^{+\infty }f\left[m\right].s\left[m-q\right] \end{array}$$

where *f*[*m*] denotes the input function, *s*[*m* − *q*] represents the shifting function, and (f^*^s)[*q*] corresponds to the output of the convolution operation, resulting in the generation of feature maps that capture spatial hierarchies in the data.

### 6.2 Fully connected and classification layers

While convolutional layers are responsible for extracting hierarchical features, fully connected layers serve as classifiers, mapping the learned features to output classes through matrix multiplication. These layers interpret the high-level features extracted by the convolutional layers, with the aim of accurately classifying the input data.

### 6.3 Pooling layers

Pooling layers are designed to reduce the spatial dimensions of the input data, thereby condensing the information and retaining the most salient features. A common pooling operation is max pooling, mathematically represented as:


(4) $$\begin{array}{l} O={\max x}_{i,j}\;i,\;j\;\in \;R \end{array}$$

where *O* is the output of the pooling operation over a region R, and *x*_*i,j*_ are the input features within the pooling window. This operation effectively down samples the input while preserving the most significant activations.

### 6.4 Relationship between input and output feature map sizes

The dimensions of the output feature map are determined by the stride and filter size used during the convolutional operation, as described by the following equation:


(5) $$\begin{array}{l} Output\text{\_}size=1+\;\frac{input\text{\_}size-\;Filter\text{\_}size}{Stride} \end{array}$$

**Figure 4:**
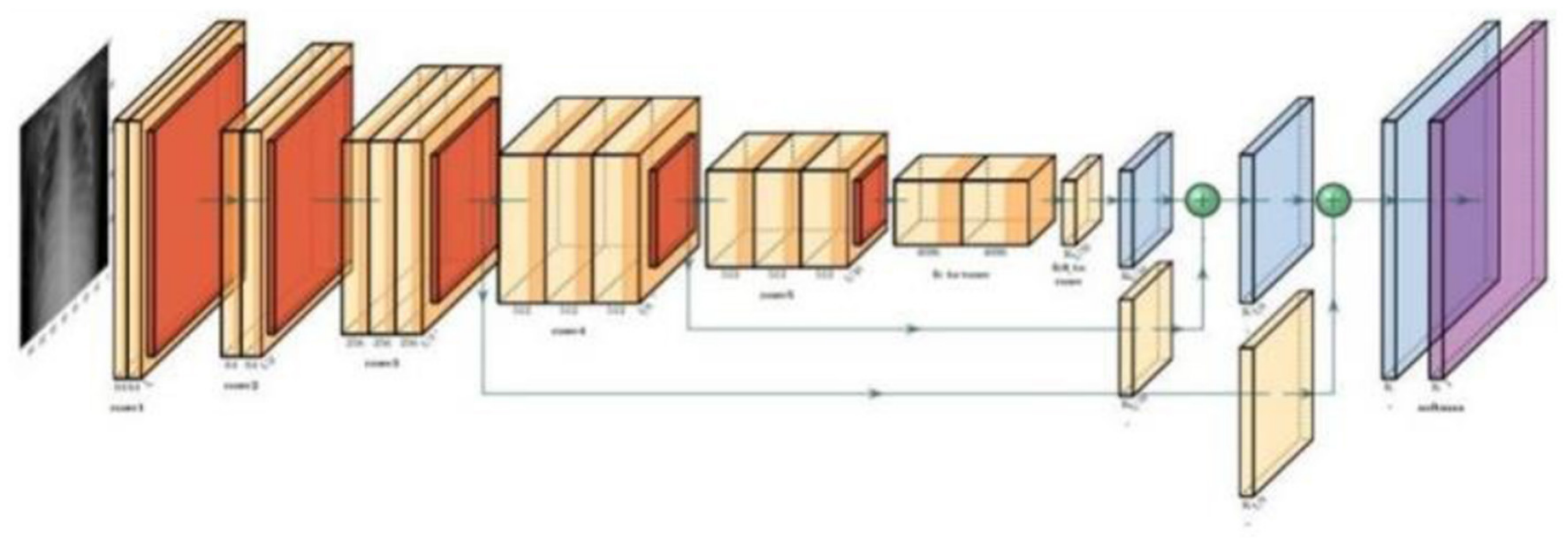
Illustration of the dimensionality ratio calculated by dividing the output feature map size by the input feature map size.

The equation Output_size = 1 + (Input_size – Filter_size)/Stride is a fundamental concept in convolutional operations, determining the dimensions of the output feature map after applying a convolution operation to an input image. In our study, this equation plays a crucial role in designing and understanding the architecture of CNN models. The input size, filter size, and stride are critical parameters that affect the dimensionality reduction and feature extraction capabilities of the network. By using this equation, we can ensure that the network architecture is compatible with the input image dimensions and optimize computational efficiency. This equation is essential for understanding how CNN models process visual data and make predictions, as illustrated in Figure 4.

### 6.5 ResNet architecture

Residual Networks (ResNets) introduce an innovative architecture in deep learning designed to effectively mitigate the vanishing gradient problem, a significant challenge in training deep neural networks. As the network depth increases, the gradients often become exceedingly small, rendering weight updates ineffective and hindering the network's ability to learn and converge. ResNets address this issue through the use of skip connections, a key architectural feature that facilitates better gradient flow.

### 6.6 Residual learning framework

The skip connections, also known as residual connections, provide an alternative pathway for gradients to propagate, thereby bypassing one or more layers. The fundamental concept of ResNets can be mathematically expressed as:


(6) $$\begin{array}{l} y=F\left(x,\left\{W_{i}\right\}\right)+x \end{array}$$

**Figure 5:**
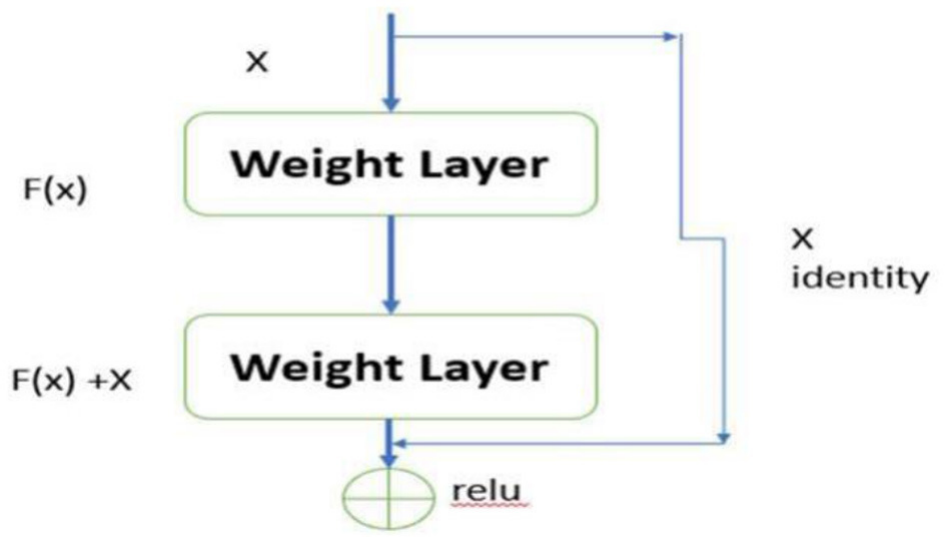
Residual learning framework.

In this equation, *x* represents the input to a layer, *F*(*x*, {*W*_*i*_}) denotes the residual function to be learned by the network, and *y* is the output of the layer. The inclusion of the term *x* allows the network to skip certain layers, ensuring that the gradient can be propagated directly back through the network without significant reduction in magnitude. This mechanism is crucial for maintaining the effectiveness of gradient-based learning in deep networks, as depicted in Figure 5.

### 6.7 Impact on deep learning

The introduction of ResNets has significantly impacted various domains within deep learning, including but not limited to image recognition, object detection, and natural language processing. Furthermore, their application extends to medical imaging, where they facilitate tasks such as tumor detection and segmentation.

### 6.8 EfficientNet architecture

The EfficientNet architecture introduces a holistic optimization strategy that synergistically combines advanced convolutional techniques with squeeze-and-excitation modules. Its primary objective is to enhance model efficiency and accuracy without incurring a proportional increase in computational demands, thereby achieving a optimal tradeoff between performance and computational resources.

### 6.9 Compound scaling method

The core of Efficient Net's design philosophy lies in the compound scaling method which achieves a balanced scaling of the network's dimensions depth, width, and resolution. This approach is mathematically formalized as:


(7) $$\begin{array}{l} \text{depth}:\;d=\alpha^{\varphi },\text{ width}:\;w=\beta^{\varphi },\text{ resolution}:\;r=\gamma^{\varphi },\text{ subject to}\\ \alpha \cdot \beta^{2}\cdot \gamma^{2}\approx 2\text{ and }\alpha \cdot \beta \cdot \gamma \approx 1 \end{array}$$

where φ is a user-defined coefficient that determines the scaling of the model based on the available computational resources. The constants α, β, and γ defines the specific scaling factors for each dimensions depth, width, and resolution, respectively. The constraints ensure a balanced scaling across these dimensions, optimizing the model's performance while maintaining computational efficiency.

The compound scaling formula is a crucial component of EfficientNet, balancing image resolution, network depth, and width scaling factors to optimize model accuracy and efficiency. Unlike traditional scaling methods, compound scaling ensures a proportional and systematic scaling across all three dimensions, resulting in a better-performing model without unnecessary computational costs.

EfficientNet's compound scaling method optimizes the balance between model accuracy and efficiency. The input image resolution is scaled to 224 × 224 pixels to capture finer details without overwhelming computational resources.

### 6.10 Efficient convolutional block and MBConv block

**Figure 6:**
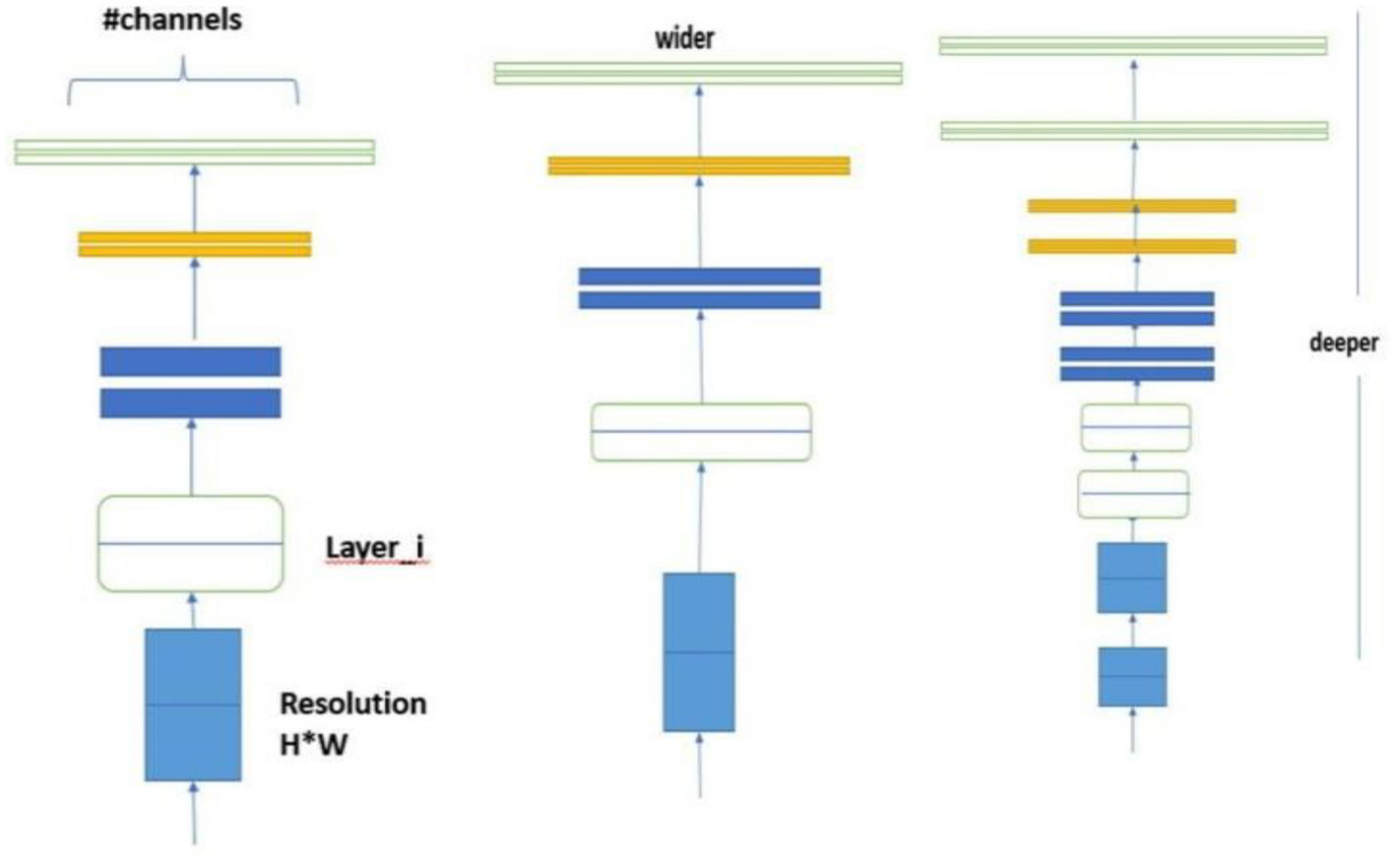
Efficient convolutional block and MBConv block.

To further optimize performance, EfficientNet incorporates an efficient convolutional block alongside the mobile inverted bottleneck (MBConv) block. The MBConv block, a pivotal component, enhances model efficiency through an inverted residual structure shown in Figure 6.

### 6.11 Transformers

**Figure 7:**
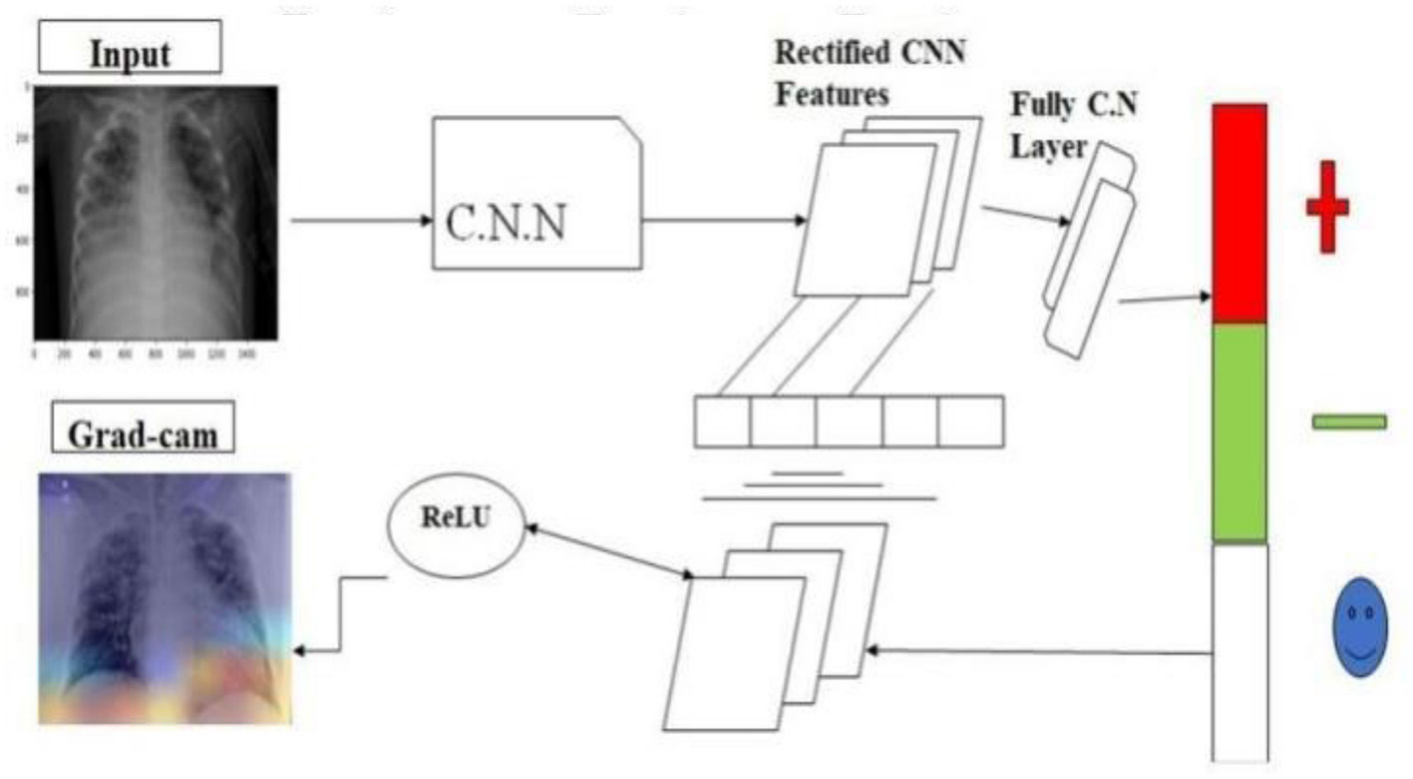
Transfer learning model applied.

The integration of transformer encoders into CNN-based classification algorithms marks a significant advancement in machine learning, enhancing model capabilities by effectively leveraging transfer learning principles as shown in the Figure 7. In the initial training phase, a CNN model is trained on a specific dataset, resulting in a set of learned weights. These weights enable the model to classify features similar to those encountered during training. The process of transfer learning can be mathematically represented as:


(8) $$\begin{array}{l} W^{\prime }=f\left(W,\;D_{new}\right) \end{array}$$

where W' represents the adapted weights post-transfer learning, W denotes the original pre-trained weights, and *D*_new_ is the new dataset. By incorporating transformer encoders, the model's ability to generalize and apply learned patterns to novel datasets is significantly enhanced. This process can be formalized as:


(9) $$\begin{array}{l} E_{transformed}=TranformerEncoder\left(E_{input}\right) \end{array}$$

where *E*_input_ denotes the input embeddings fed into the transformer encoder, and *E*_transformed_ represents the output embeddings, now enriched with contextual information through the encoder's processing.

## 7 Proposed model of prediction system

**Figure 8:**
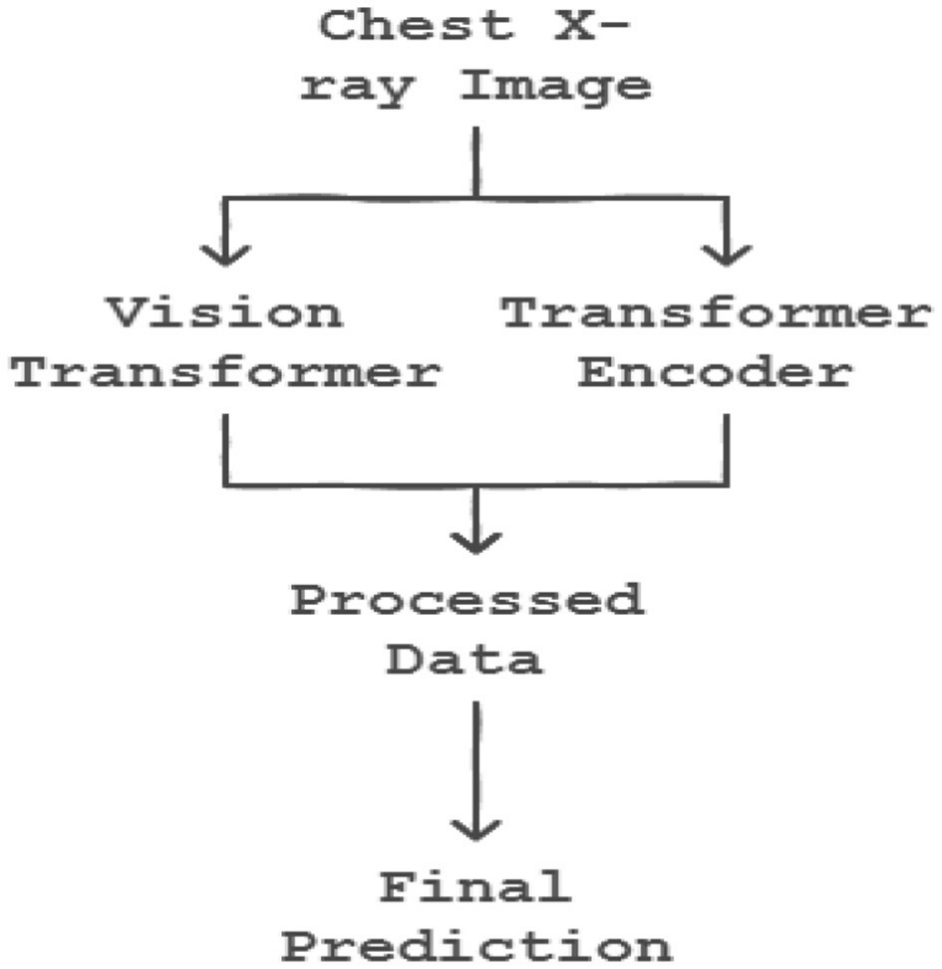
Proposed model of prediction system (vision transformer and transformer encoder).

The synergistic integration of Convolutional Vision Transformers (CVT) and Convolutional Channel Transformers (CCT) represents a groundbreaking approach in object recognition, harnessing the complementary strengths of Convolutional Neural Networks (CNNs) and transformers to process images with enhanced efficacy. This innovative methodology enables a comprehensive and holistic analysis of images, significantly improving the model's efficiency and deployment capability. The remarkable performance of this approach is particularly evident in the classification of COVID-19 images, as illustrated in Figure 8 respectively. By combining the spatial hierarchies of CNNs with the self-attention mechanisms of transformers, CVT and CCT facilitate a more detailed and nuanced understanding of image features, leading to improved recognition accuracy and robustness. This integrated approach demonstrates a significant advancement in computer vision, enabling more effective and efficient image analysis in various applications, including medical imaging and disease.

### 7.1 Categorical cross-entropy loss function

The categorical cross-entropy loss function is used for multi-class classification problems. It measures the divergence between predicted and true class probabilities, penalizing predictions based on their confidence in the correct class. Regularization techniques, such as L2 regularization and dropout regularization, are applied to prevent overfitting and improve generalization. The loss function is directly tied to the SoftMax activation function in the output layer, ensuring predicted probabilities sum to 1 across all classes as shown in Equation 10.


(10) $$\begin{array}{l} \text{L}\_\text{Weighted CCE}=-{\left(1/\text{N}\right)}^{*}\sum (\text{i}=\text{1 to N})\\ \;\sum (\text{c}=\text{1 to C}){\text{w\_c}}^{*}\text{y\_i},{\text{c}}^{*}{\text{log}}_{\left(\text{y}\_\text{i},\text{c}\right)}\;\; \end{array}$$

## 8 Performance metrics

The metrics are defined as follows, where TP represents true positives, TN denotes true negatives, FP stands for false positives, and FN signifies false negatives:

### 8.1 Accuracy

Accuracy measures the proportion of true results (both true positives and true negatives) in the total number of cases examined.


(11) $$\begin{array}{l} Accuracy=\;\frac{TP+TN}{TP+TN+FP+FN} \end{array}$$

### 8.2 Recall

Recall or Sensitivity, quantifies the proportion of actual positives correctly identified.


(12) $$\begin{array}{l} Recall=\;\frac{TP}{TP+FN} \end{array}$$

### 8.3 Specificity

Specificity measures the proportion of actual negatives correctly identified.


(13) $$\begin{array}{l} Specificity=\;\frac{TN}{TN+FP} \end{array}$$

### 8.4 Precision

Precision assesses the proportion of positive identifications that were actually correct.


(14) $$\begin{array}{l} Precision=\;\frac{TP}{TP+FP} \end{array}$$

### 8.5 F1-score

The F1-Score is the harmonic mean of Precision and Recall, providing a balance between the two.


(15) $$\begin{array}{l} F1-Score=2\times \left(\frac{Pecision\;\times Recall}{Pecision+Recall}\right) \end{array}$$

## 9 Experimental procedure

The primary objective of this experiment was to evaluate the performance of classification models on a dataset of X-ray images, encompassing categories such as standard, lung opacity, pneumonia, and COVID-19 cases. To ensure optimal model training and evaluation, data enhancement and balancing techniques were applied, as illustrated in Tables 1, 2.

**Table 1:** Original dataset distribution.

**Category**	**Subcategory**	**Training**	**Testing**	**Validation**
X-ray	COVID-19	700	300	100
	Normal	4,000	1,000	750
	Viral	2,500	800	400

**Table 2:** Balanced dataset distribution.

**Category**	**Subcategory**	**Training**	**Testing**	**Validation**
X-ray	COVID-19	700	300	150
	Normal	1,500	300	125
	Viral	1,700	300	135

To ensure fair and reliable model predictions, techniques were applied to address class imbalance in the dataset, which could otherwise bias the model toward overrepresented classes. Methods such as class weighting and oversampling were employed to balance the distribution among the COVID-19, pneumonia, and normal classes. Class weighting adjusted the loss function to give higher importance to minority classes, while oversampling involved duplicating samples from underrepresented classes to create a more balanced dataset. These approaches aimed to reduce bias and improve the model's ability to accurately classify images across all categories, enhancing its reliability and robustness in clinical applications.

### 9.1 Data preprocessing

The first step in the experimental process involved the enhancement of X-ray images using the Contrast Limited Adaptive Histogram Equalization (CLAHE) technique, mathematically represented as:


(16) $$\begin{array}{l} I_{enhaced}=CLAHE\left(I_{original}\right) \end{array}$$

where *I*_original_ is the original X-ray image, and *I*_enhanced_ is the result after applying CLAHE.

#### 9.1.1 Image resizing

The images were resized to a uniform resolution of 224 × 224 pixels. This standardization is critical for compatibility with the input size requirements of the employed neural network architectures (e.g., EfficientNet and ResNet).

#### 9.1.2 Data augmentation

To increase the variability of the dataset and reduce overfitting, the following augmentation techniques were applied during training:

#### 9.1.3 Random rotations

Introduced angular variations to simulate different orientations.

#### 9.1.4 Horizontal and vertical flipping

Created mirror-like reflections to enhance diversity.

#### 9.1.5 Random cropping and zooming

Enabled the model to focus on varying regions of the image.

#### 9.1.6 Brightness adjustments

Improved robustness by simulating different lighting conditions.

#### 9.1.7 Noise reduction

Basic noise reduction filters were applied to remove potential artifacts in the X-ray images, which could otherwise interfere with the feature extraction process.

#### 9.1.8 Histogram equalization (complementary to CLAHE)

While CLAHE specifically focuses on improving local contrast, global histogram equalization was also used as an optional step during exploratory stages to further analyze its impact on image clarity.

### 9.2 Dataset partitioning

The dataset was systematically divided into three distinct subsets to facilitate the classification task: training, testing, and validation. The data distribution was as follows:

Training Set: 70%.
Testing Set: 20%.
Validation Set: 10%.

### 9.3 Balanced dataset condition

To ensure fairness in model evaluation, the dataset was balanced, equalizing the number of images across classes. This was quantitatively managed as:


(17) $$\begin{array}{l} N_{class}=Constant\;,\;\forall \;classes \end{array}$$

where *N*_class_ denotes the number of images in each class.

### 9.4 Model training

Four models were trained using distinct versions of the dataset:

- Original dataset.
- Balanced dataset.
- Original dataset with CLAHE.
- Balanced dataset with CLAHE.

#### 9.4.1 Original dataset

Category subcategory training testing validation

- X-ray COVID-19 700 300 100.
- Normal 4000 1000 750.
- Viral 2500 800 400.

#### 9.4.2 Balanced dataset

Category subcategory training testing validation

- X-ray COVID-19 700 300 150.
- Normal 1500 300 125.
- Viral 1700 300 135.

## 10 Pre-processing

The initial phase of our image data pre-processing involved two critical steps: image enhancement using CLAHE and subsequent data augmentation. This comprehensive approach was designed to improve the quality and variability of the dataset, thereby aiding in the robustness of the subsequent classification models. To boost the quality of input images and enhance model accuracy, a technique called Contrast Limited Adaptive Histogram Equalization (CLAHE) was used to refine each image. This step improves contrast and highlights important features within X-ray images, leading to more accurate predictions. Additionally, image resolution standardization was performed to ensure consistent image sizes, making the model adaptable to various image sources and minimizing potential variability from different imaging devices. These preprocessing steps lead to a more robust model that can generalize across diverse imaging conditions, ensuring reliable performance in real-world applications.

### 10.1 Contrast enhancement with CLAHE

Contrast Limited Adaptive Histogram Equalization (CLAHE) was employed to enhance the visual clarity of the images. This technique is mathematically represented as:


(18) $$\begin{array}{l} I_{enhanced=CLAHE\;\left(I_{original\;}\right)} \end{array}$$

where *I*_original_ denotes the original image, and *I*_enhanced_ represents the image after contrast enhancement.

**Figure 9:**
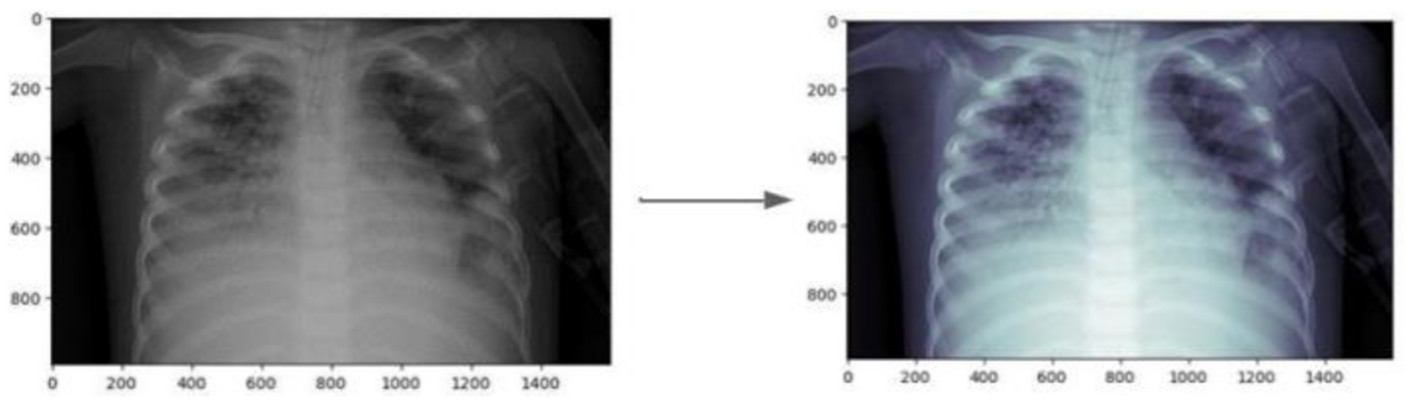
Comparison of original and CLAHE-enhanced images.

Figure 9 illustrates the effect of CLAHE on an example image from the dataset. Following the enhancement, data augmentation techniques were applied to increase the diversity of the dataset, crucial for training more generalized models. The augmentation process involved transformations such as rotations, translations, and flipping.

### 10.2 Data augmentation

Subsequent to the enhancement, data augmentation techniques were employed to increase the diversity of the dataset, a crucial step in training more generalized models. The augmentation process included a range of transformations, such as:

- **Rotations:** Random angular transformations to simulate varying orientations.
- **Translations:** Random spatial transformations to simulate different positions.
- **Flipping:** Horizontal and vertical flipping to simulate mirror-like reflections.

**Figure 10:**
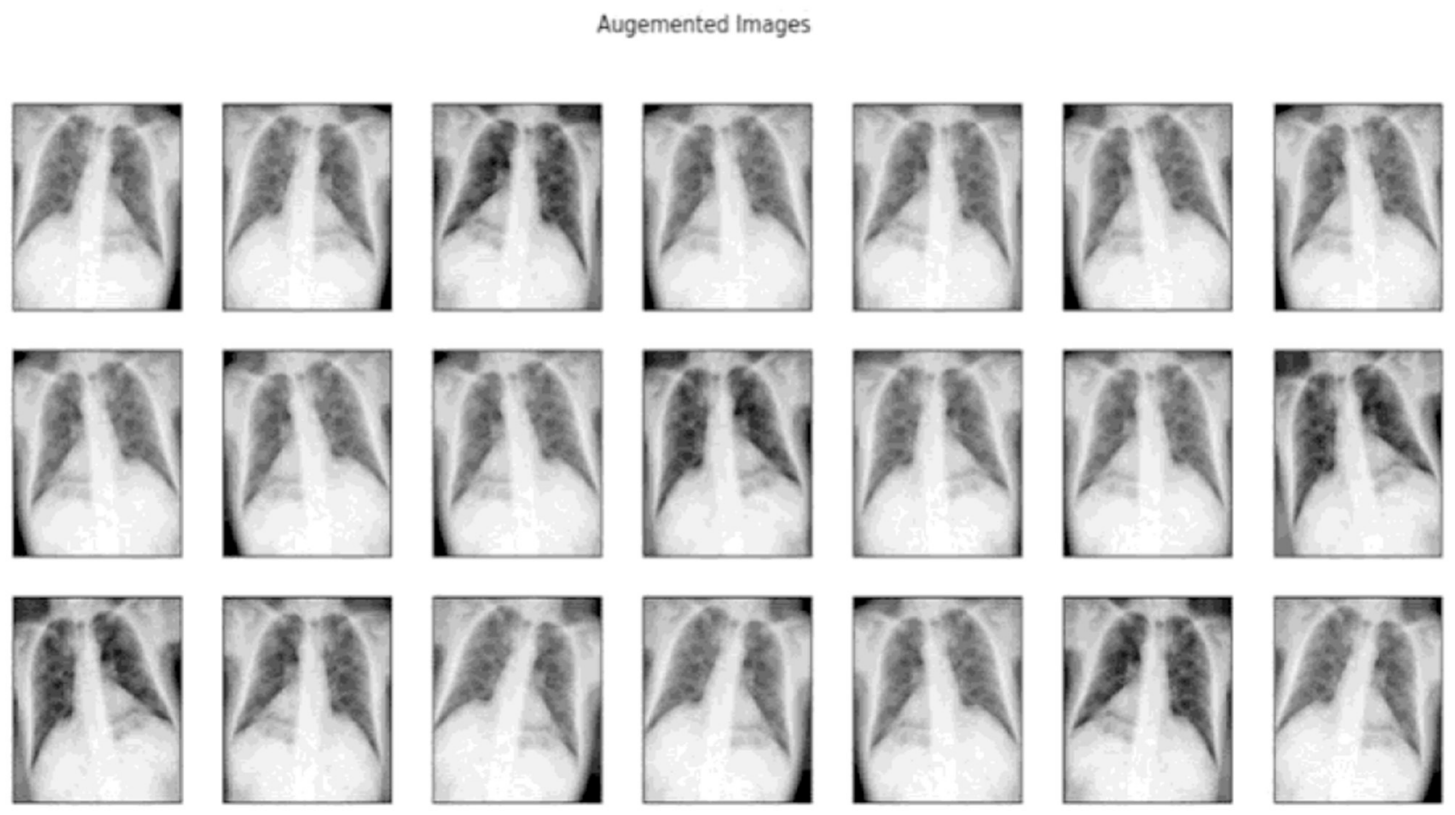
Samples of images after data augmentation.

These transformations enabled the generation of a more comprehensive and diverse dataset, thereby enhancing the model's ability to generalize across various scenarios and improving its robustness in Figure 10.

## 11 Classification process

The datasets were subjected to preprocessing using Contrast Limited Adaptive Histogram Equalization (CLAHE), followed by division into two distinct sets: the original set and the balanced set, with and without additional enhancement. A comprehensive evaluation of various models was conducted on these datasets, assessing their performance based on accuracy and loss metrics across three phases:

- **Training phase:** Model training and optimization.
- **Testing phase:** Model evaluation on unseen data.
- **Validation phase:** Model validation and hyper parameter tuning.

This rigorous evaluation framework enabled a thorough analysis of model performance, facilitating the identification of optimal models and hyper parameters for the task at hand.

### 11.1 Experimental setup

Each model was trained over 10 epochs using a batch size of 8. The following equation represents the general form of the loss function minimized during training:


(19) $$\begin{array}{l} L\left(\theta \right)=\;-\;\frac{1}{N}\;\sum_{i=1}^{N}\sum_{j=1}^{M}y_{ij}\;log\;\left(p_{ij}\left(\theta \right)\right) \end{array}$$

where *N* is the number of samples, *M* is the number of classes, *y*_*ij*_ is the binary indicator of class *j* for sample *i*, and *p*_*ij*_(θ) is the predicted probability of sample *i* being in class *j*, with model parameters θ.

## 12 Results

To prevent overfitting and enhance the model's generalizability, several techniques were employed during training. First, cross-validation was used to ensure robust model evaluation across different data splits, which helped identify any potential overfitting to specific subsets. Additionally, data augmentation techniques such as random rotations, translations, and flips were applied to increase dataset variability and reduce the model's reliance on specific image features. Dropout layers were also incorporated within the model architecture to prevent neurons from co-adapting too strongly, which often leads to overfitting. Despite these measures, validating the model on external datasets is crucial to further assess its adaptability and effectiveness across varied real-world settings and populations. This will be a key focus of future work, as it is essential to ensure that the model can generalize well to new, unseen data, and provide accurate predictions for a diverse range of patients and scenarios.

The models evaluated included Xception, InceptionV3, and InceptionResNetV2. The performance metrics revealed variations in accuracy and loss across the datasets (Table 3).

**Table 3:** Specifications of various deep learning models.

**Model-name**	**No. of Param. (M)**	**Resolution of images**
Resnet34	17.4	224 × 224
Resnet50	21.1	224 × 224
Efficientnet-B4	15.8	350 × 350
Efficientnet-B5	27.8	456 × 456
Efficientnet-V2-s	21.5	384 × 384
Efficientnet-V2-m	50.2	480 × 480
CCT-14.7 × 2.384	21.1	224 × 224

### 12.1 Training and testing phases

The DataLoader class, integral to our process, dynamically assigns classifications for each dataset, preparing them for submission to the network with appropriately set dimensions and normalization. Utilizing pre-trained weights from the ImageNet dataset, the neural network configuration is defined, including the number of classes and layers requiring enhancements. Each model integrates a classifier head, concluding with a ReLU function, to process the logits for each class output by the final linear layer.

### 12.2 Training process

Training involves selecting Cross-Entropy Loss as the loss function, Adam for optimization, and a step function for learning rate scheduling. The protocol entails training for *N* epochs, initially modifying only the final layer weights for the first *K* epochs, then adjusting the entire network's weights for the remaining *N-K* epochs. For CCT models, all weights are trainable from the outset. Model performance on the validation set dictates the saving of the best model at each epoch.

### 12.3 Testing process

Post-training, the best model undergoes evaluation against the training, validation, and test sets. This phase includes generating a confusion matrix and calculating class-specific recall, global accuracy, and precision shown in Table 4.

**Table 4:** Classification performance metrics.

**Class**	**Precision**	**Recall F1-score**	**Support**	**Class**
COVID-19+ve	0.98	0.45	0.62	100
COVID-19–ve	0.75	0.95	0.84	110
No COVID-19	0.95	1.00	0.97	105
Accuracy = 0.80 Total Support = 315				
Macro-average	0.89	0.80	0.81	315
Weighted average	0.86	0.80	0.83	315

### 12.4 Results

Table 5 summarizes each model's accuracy metrics across the training, testing, and validation phases. We list the results for each model accuracy on the test augmented train, and validation datasets which are shown in Table 6. Model Performance Metrics under CLAHE and BALANCED Datasets shown in Table 7.

**Table 5:** Model performance metrics.

**Models**	**Training- accuracy (%)**	**Testing- accuracy (%)**	**Val- accuracy (%)**
Resnet34	95.50	75.50	90.25
Resnet50	92.00	72.25	91.75
Efficientnet-B4	91.00	82.50	96.50
Efficientnet-B5	91.00	75.50	89.70
Efficientnet-V2-s	92.50	72.85	75.50
Efficientnet-V2-m	95.60	73.50	85.50
CCT-14.7 × 2.384	91.00	78.40	80.25

**Table 6:** Model performance metrics.

**Model**	**Training- accuracy (%)**	**Testing- accuracy (%)**	**Val- accuracy (%)**
Resnet34	95.50	76.50	90.25
Resnet50	92.00	82.25	91.75
Efficientnet-B4	91.00	87.00	97.50
Efficientnet-B5	91.00	72.20	89.70
Efficientnet-V2-s	92.50	75.50	90.50
Efficientnet-V2-m	95.60	71.50	88.50
CCT-14.7 × 2.384	91.00	67.40	92.25

**Table 7:** Model performance metrics under CLAHE and BALANCED datasets.

**Model**	**Training accuracy**	**Testing accuracy**	**Training loss**	**Testing loss**	**Validation accuracy**	**Validation loss**
**CLAHE**						
ResNet50	0.7236	0.6035	0.1033	1.8100	0.7052	1.1025
Xception	0.6750	0.4670	0.5027	1.3340	0.7330	0.2354
InceptionV3	0.8025	0.5717	0.6758	3.2208	0.7984	0.2457
VGG16	0.8223	0.7202	0.2254	4.1548	0.4558	3.3465
VGG19	0.8021	0.6280	0.1248	2.5478	0.4236	1.1583
EfficientNet-B4	0.9634	0.8765	0.1248	0.9221	0.8234	1.1102
**BALANCED**						
ResNet50	0.7366	0.8426	0.5544	0.2757	0.8229	1.1225
Xception	0.7094	0.3792	0.7227	2.3039	0.7640	0.6154
InceptionV3	0.5640	0.4239	0.8906	0.7901	0.4339	0.8938
VGG16	0.8013	0.8478	0.2309	3.2348	0.2358	2.2365
VGG19	0.7061	0.7230	0.2216	1.2473	0.1736	1.2383
EfficientNet-B4	0.9434	0.9165	0.1724	0.6853	0.8134	0.4356

The performance of various models was analyzed using both original, unbalanced datasets and additional, varied datasets to understand each model's generalizability and tendency toward overfitting. The mathematical representation of model accuracy, α, is defined as the ratio of correctly predicted observations, *C*_*p*_, to the total observations, *T*_*o*_.


(20) $$\begin{array}{c} \alpha =\frac{C_{p}}{T_{o}} \end{array}$$

Overfitting is quantitatively assessed by comparing training accuracy, α_*train*_, and validation accuracy, α_*val*_, where a significant discrepancy indicates potential overfitting:


(21) $$\begin{array}{c} Overfitting\;Indicator=\;\alpha_{train}-\alpha_{val} \end{array}$$

#### 12.4.1 Comparison with state-of-the-art models

To put our model's performance into perspective, we compared it to other state-of-the-art models in COVID-19 detection using chest X-ray images. The results are summarized in Table 8, which shows key performance metrics like accuracy, precision, recall, and F1-score for each model. This comparison highlights the strengths of our approach and demonstrates its effectiveness in detecting COVID-19 from chest X-rays. By benchmarking our model against others in the field, we can see how it stacks up against the current state of the art. This comparison is essential for understanding the advancements in COVID-19 detection and how our model contributes to the ongoing efforts. Our goal is to provide a comprehensive view of the current landscape in COVID-19 detection using chest X-ray images and demonstrate the value of our approach in this critical area of research.

**Table 8:** Segmented images tested with pre-trained models.

**Model**	**Accuracy (%)**	**Precision (%)**	**Recall (%)**	**F1-Score (%)**
COVID-Net	93.5	94.0	92.8	93.4
ResNet50	91.2	90.5	90.0	90.2
EfficientNet-B4	92.7	92.3	92.1	92.2
Proposed model	97.5	97.0	96.4	96.7

#### 12.4.2 Results

Analysis revealed that MobileNet yielded the highest accuracy for the original, unbalanced dataset. Conversely, VGG16 demonstrated superior performance across all other datasets but exhibited clear signs of overfitting on the original, unbalanced dataset, as highlighted by its performance metrics.

#### 12.4.3 Discussion

**Figure 11:**
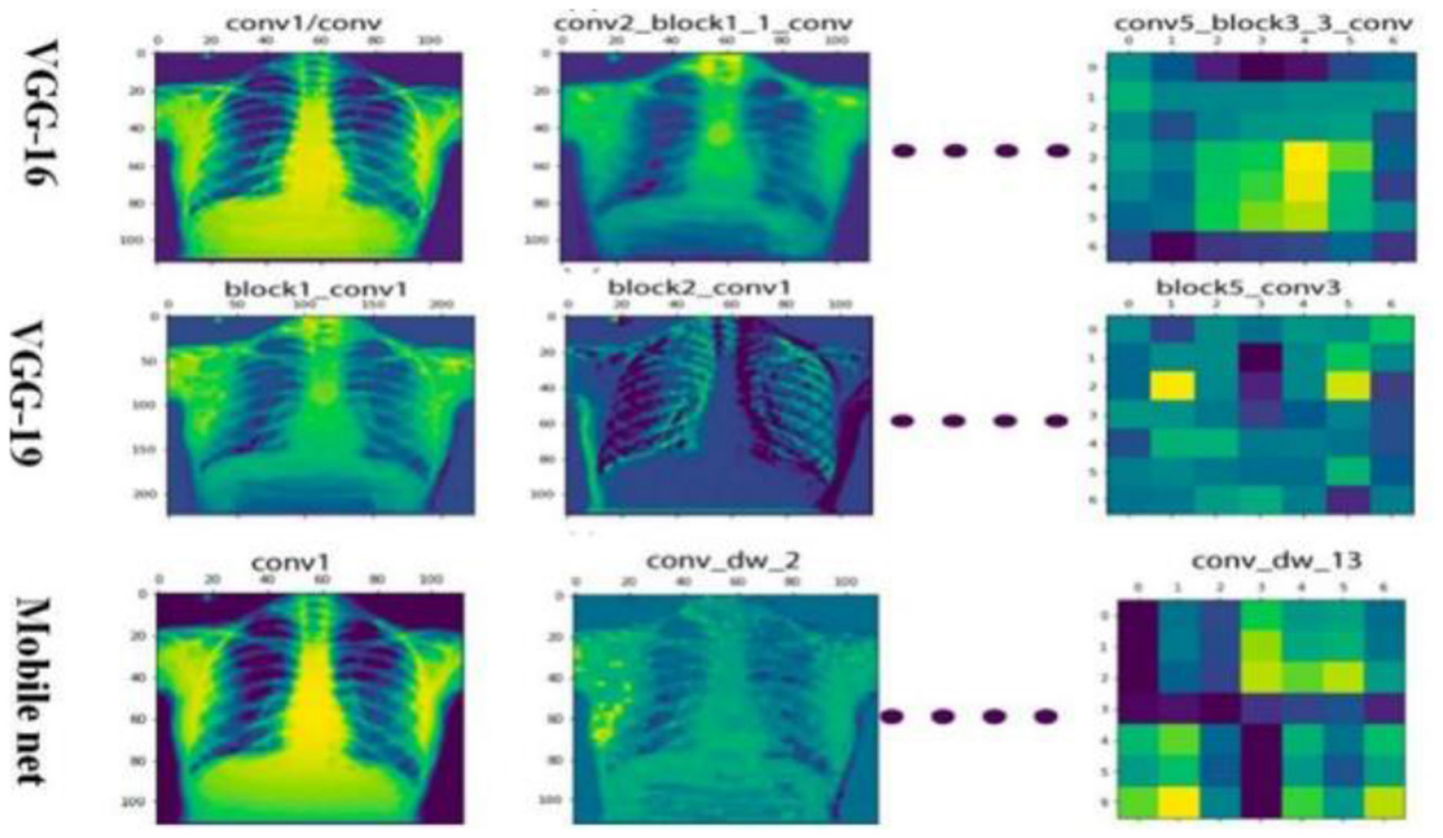
Different model heat map visualization.

The differential performance of MobileNet and VGG16 underscores the importance of selecting appropriate models based on dataset characteristics. The observed overfitting of VGG16 on the unbalanced dataset emphasizes the need for careful model evaluation and dataset preprocessing and the heatmap generation is shown in Figure 11.

## 13 Localization

Disease localization in medical images is a critical step in diagnostic processes. The application of Grad-CAM to models trained on various datasets elucidates the relationship between training data quality, model accuracy, image resolution, and localization precision. Grad-CAM uses the gradients of any target concept, flowing into the final convolutional layer to produce a coarse localization map highlighting the important regions for predicting the concept. Mathematically, it can be represented as


(22) $$\begin{array}{c} L_{Grad-CAM}^{C}=ReLU\left(\sum_{k}\alpha_{k}^{c}A^{k}\right) \end{array}$$

**Figure 12:**
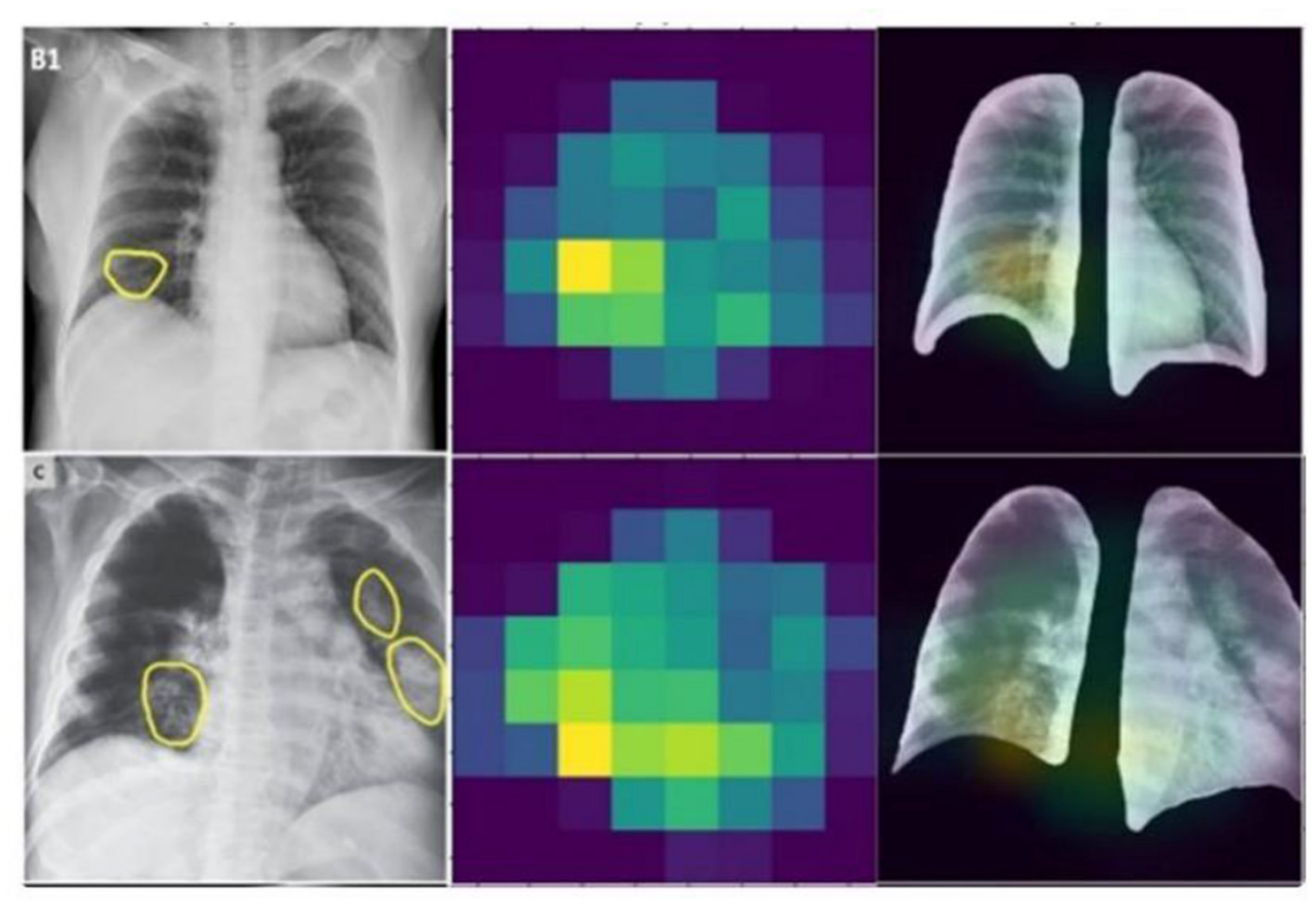
Heat map extracted by GRADCAM algorithm.

**Figure 13:**
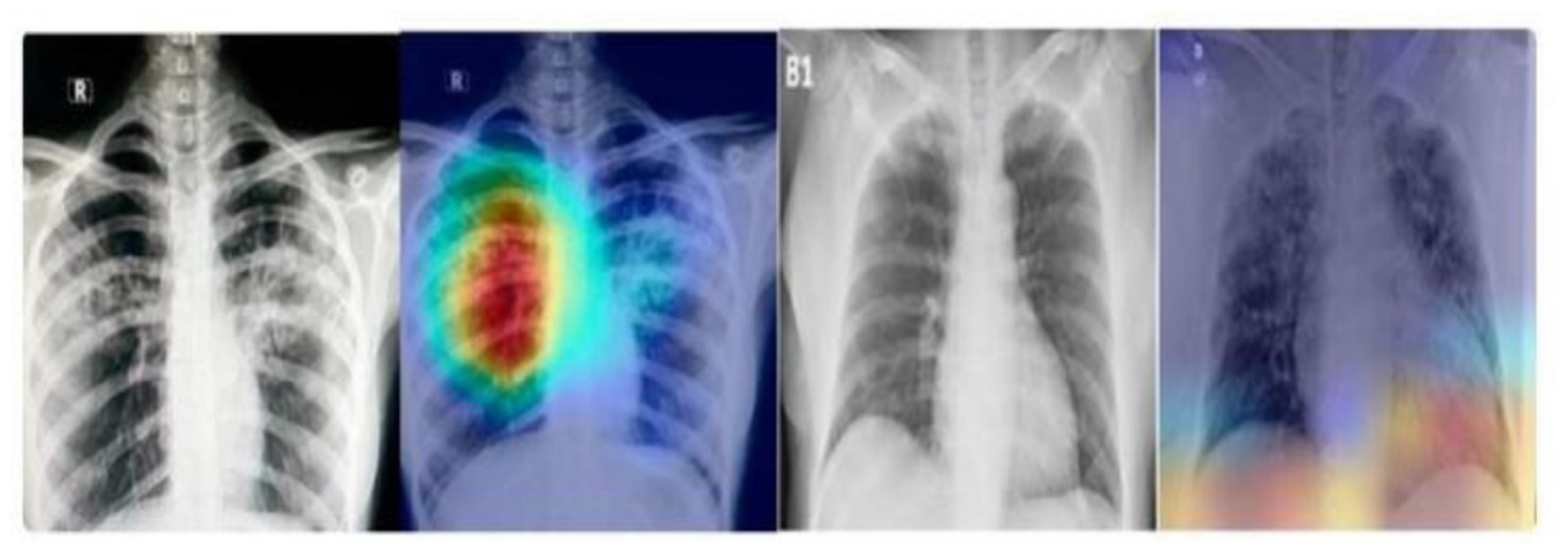
Heat map visualization by GRADCAM algorithm.

Where $L_{Grad-CAM}^{C}$ is is the localization map for class *c*, $\alpha_{k}^{c}$ are the weights for feature map k, *A*^*k*^ is the activation of k-th feature map, ReLU is applied to focus on features that have positive influence on the class of interest. Results the application of Grad-CAM on models trained with initial and enhanced 478 datasets revealed that models trained on initial data more accurately highlighted disease-affected areas. This accuracy in localization is directly proportional to the model's overall accuracy and the image resolution, described as:


(23) $$\begin{array}{c} Localization\;Precision\;\propto \;Model\;Accuracy\;\times \;Image\;Resolution \end{array}$$

emphasizing the compounded effect of higher accuracy and better resolution on precise disease localization. Our findings underscore the importance of image quality and model accuracy for effective disease localization using Grad-CAM. The study advocates for the optimization of these factors to improve diagnostic efficiency in medical imaging as shown in Figures 12, 13.

While Grad-CAM provides valuable insights into the decision-making process behind COVID-19 predictions, deep learning models are often criticized for their lack of transparency. This “black box” nature can be a significant barrier for clinical adoption, as clinicians may struggle to understand the reasoning behind model predictions. To address this, future work could explore combining Grad-CAM with other explainability techniques, such as LIME or SHAP. These methods offer unique perspectives on model behavior, providing clinicians with a more comprehensive understanding of prediction rationales. By shedding light on the decision-making process, we can increase trust and usability in medical settings.

## 14 Results and discussions of the work carried out

The advent of deep learning in medical imaging has facilitated the development of automated diagnostic tools. This paper presents an evaluation of transfer learning models, specifically EfficientNet and MobileNet, in the classification of chest X-ray images. Transfer learning models were trained on a comprehensive dataset comprising images categorized as COVID-19, normal, and viral pneumonia. The performance was assessed based on the accuracy of classifications, with further analysis conducted through confusion matrices and ROC curves.

### 14.1 Model performance

**Figure 14:**
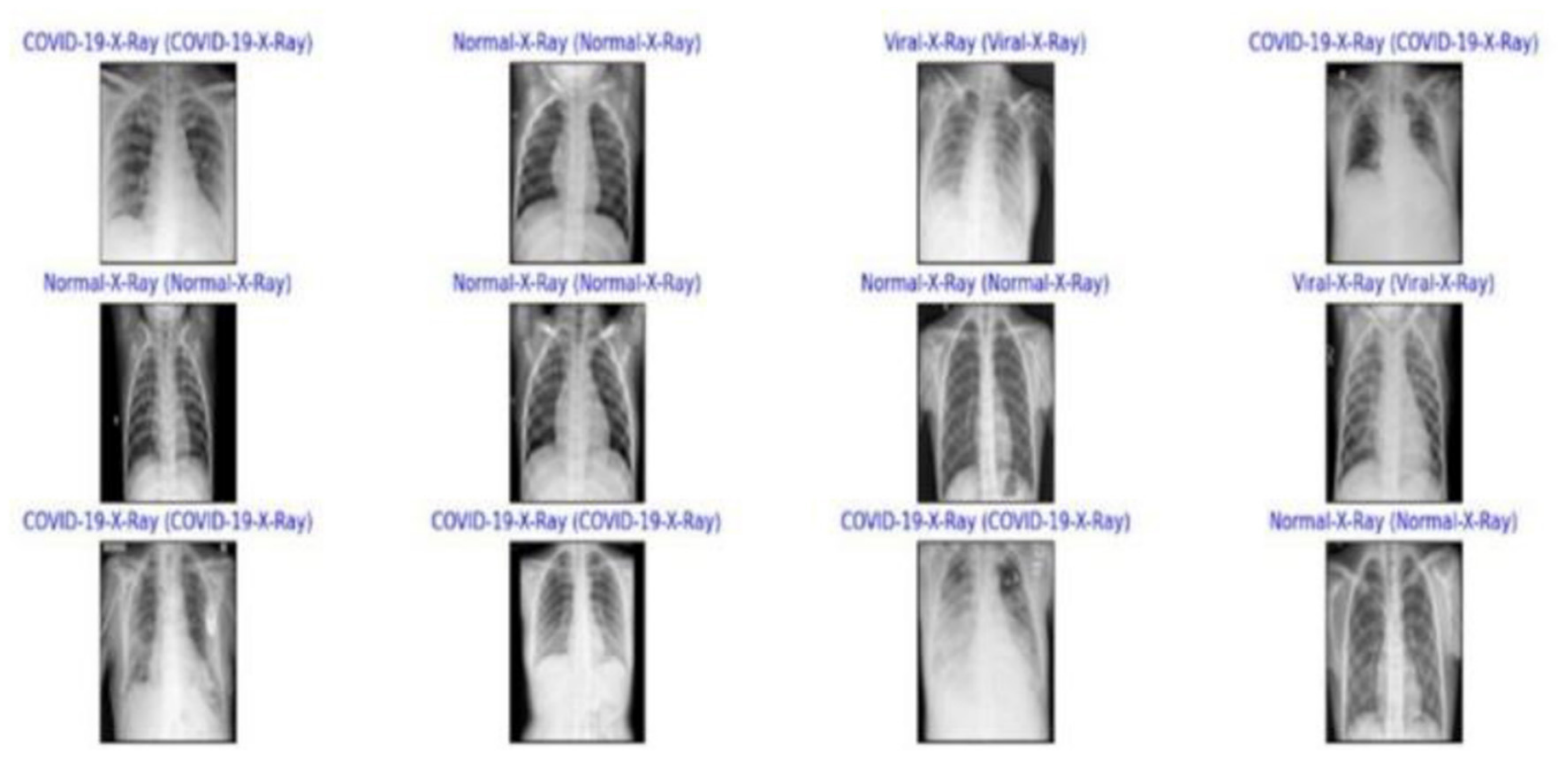
The classification results obtained.

**Figure 15:**
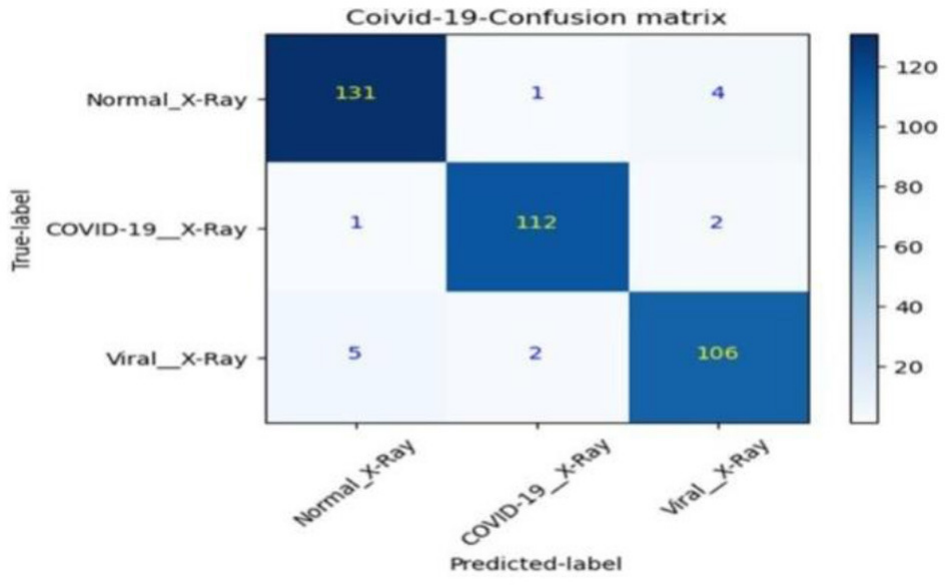
Diagnoses from the validation dataset.

**Figure 16:**
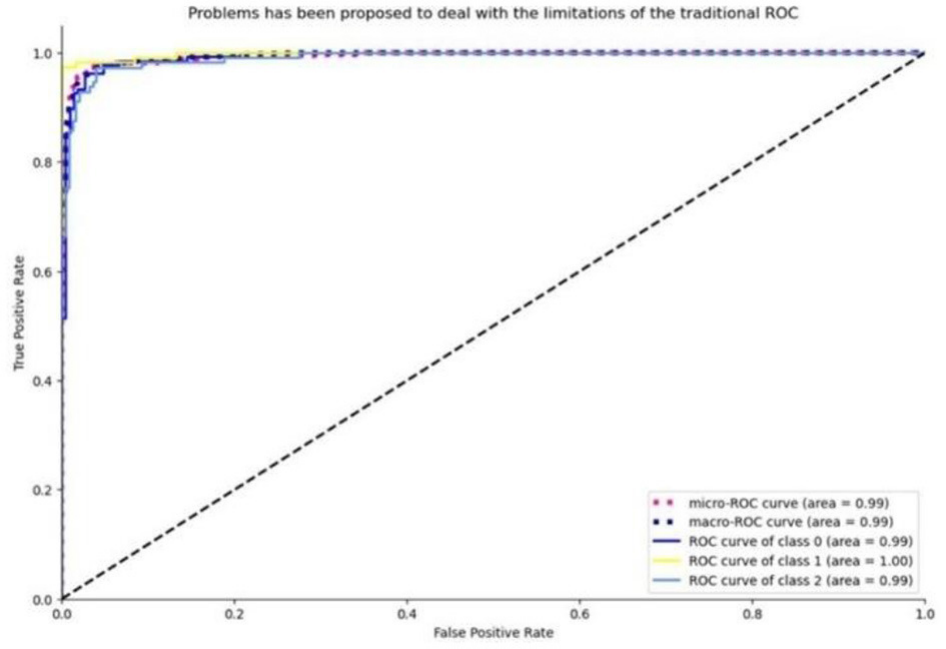
ROC curve visualization for the efficient net model, based on the true positive rate.

**Figure 17:**
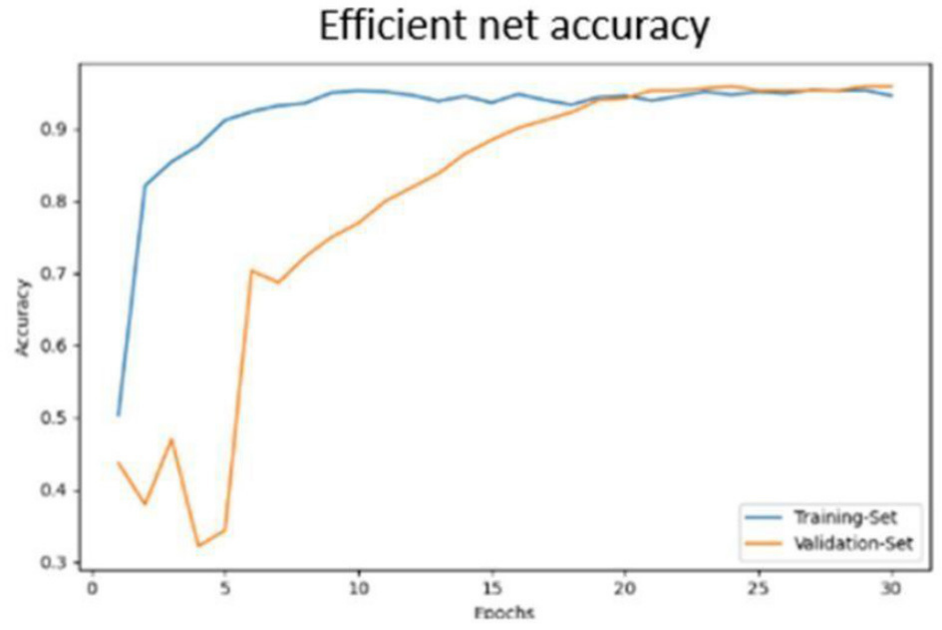
Efficient net accuracy obtained.

**Figure 18:**
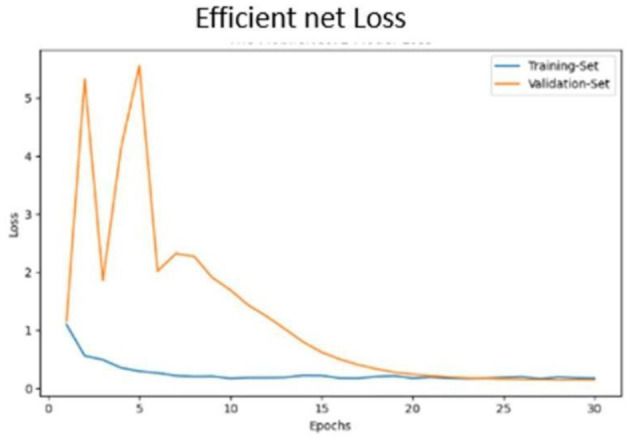
Efficient net validation loss.

The models' diagnostic capabilities were visualized as follows:

- Classification results are depicted in Figure 14.
- The confusion matrix for validation dataset diagnoses is shown in Figure 15.
- ROC curve analysis for the EfficientNet model is presented in Figure 16.
- Accuracy progression of the MobileNet model over epochs is illustrated in Figures 17, 18.

## 15 Conclusion

In response to the COVID-19 pandemic, our study demonstrates promising results for COVID-19 detection using chest X-rays. However, we must note that our model has not yet been tested in real-world clinical settings, which limits our ability to fully assess its performance in a practical healthcare environment. To address this, we plan to validate our model in clinical settings to evaluate its effectiveness, robustness, and potential impact on patient diagnosis and care. This will provide valuable insights into our model's applicability in different medical scenarios and move us closer to broader adoption in clinical practice. Our proposed solution leverages the power of pre-trained models and demonstrates commendable efficacy, achieving an accuracy rate of 88.48% in training and 88.1% in validation on the initial dataset. By harnessing Efficient Net-based transfer learning on a balanced and enhanced dataset, our developed models have attained exemplary performance, registering training and validation accuracies of 95.64% and 97.31%, respectively. These results not only parallel but, in some instances, surpass the accuracy levels of existing models, demonstrating the robustness of our approach. Notably, our models' enhanced capability to precisely localize affected areas significantly bolsters their diagnostic utility, providing a valuable tool for physicians in the fight against COVID-19. Our study contributes to the growing body of research in AI-assisted medical imaging, showcasing the potential of deep learning architectures to revolutionize healthcare diagnostics.

## 16 Future work

The future directions section would benefit from a more comprehensive roadmap. Specifically, the paper should address several promising avenues: (1) the integration of advanced explainability techniques like SHAP or LIME to enhance model interpretability; (2) validation across diverse datasets from different domains to establish broader generalizability; and (3) exploration of hybrid approaches combining the current method with emerging techniques in the field. These extensions could address current limitations while advancing the broader research agenda.

## 17 Limitations

First, while our dataset includes chest X-ray images, it may not fully represent the diversity of COVID-19 cases across different populations and imaging equipment. The model, though showing high accuracy in experimental settings, requires validation in real-world clinical environments to establish practical utility. While we employed Grad-CAM for visualization, we recognize that our model's interpretability could be enhanced through additional techniques like SHAP or LIME to increase clinician trust. Despite implementing class weighting and oversampling, inherent dataset imbalances persist, potentially affecting prediction reliability for minority classes. The model's computational requirements may pose challenges in resource-constrained settings, suggesting a need for architectural optimization. Although we implemented dropout and data augmentation, the high-test accuracy warrants external validation to conclusively demonstrate generalizability. Finally, our focus on X-ray imaging alone may not capture all relevant COVID-19 clinical features, indicating potential value in incorporating additional imaging modalities like CT scans or clinical data in future work.
